# Patient exposure during videofluoroscopic swallowing studies performed by speech-language pathologists

**DOI:** 10.1093/rpd/ncad181

**Published:** 2023-06-21

**Authors:** Ioannis A Tsalafoutas, Shady AlKhazzam, Mohammed S Hussain, Ahmed J Omar, Salwa S Bawazir, Suad A AlHamad, Huda AlNaemi, Mohammad H Kharita

**Affiliations:** Medical Physics Section, OHS Department, Hamad Medical Corporation, PO Box 3050, Doha, Qatar; Medical Physics Section, OHS Department, Hamad Medical Corporation, PO Box 3050, Doha, Qatar; Speech and Language Pathology Department, Qatar Rehabilitation Institute, PO Box 3050, Doha, Qatar; Clinical Imaging Department, Hamad General Hospital, PO Box 3050, Doha, Qatar; Clinical Imaging Department, Hamad General Hospital, PO Box 3050, Doha, Qatar; Clinical Imaging Department, Hamad General Hospital, PO Box 3050, Doha, Qatar; Medical Physics Section, OHS Department, Hamad Medical Corporation, PO Box 3050, Doha, Qatar; Medical Physics Section, OHS Department, Hamad Medical Corporation, PO Box 3050, Doha, Qatar

## Abstract

Videofluoroscopic swallowing studies (VFSSs) are fluoroscopic examinations performed by speech and language pathologists (SLPs), for the evaluation of the oral and pharyngeal phases of swallowing, in patients who are diagnosed with symptoms like dysphagia and speech impairment. The study was focused on the evaluation of the patient doses from VFSS performed at Hamad Medical Corporation hospitals. Data on the patient exposure and examination parameters were extracted from the Radiation Dose Monitoring system, statistically analysed and compared with literature. For adult patients, the mean (median) values for fluoroscopy time and kerma-air product were 2.8 (2.7) min and 181 (144) cGycm^2^, respectively. For children, the respective mean (median) values were 2.6 (2.4) min and 15.3 (9.2) cGycm^2^. The results of the study indicate that the VFSS are performed by well-trained health professionals, and as a result, image quality sufficient for a confident diagnosis is obtained at relatively low dose levels.

## Introduction

The videofluoroscopic swallowing study (VFSS), also known as modified barium swallowing study (MBSS), is a fluoroscopic examination performed for the evaluation of the oral and pharyngeal phases of swallowing, in patients which present symptoms like dysphagia and speech impairment. These symptoms result in ~50–60% of patients post-stroke^(1)^. Most of the patients with dysphagia self-improve within a period of a week and only 11–13% remains dysphagic after 6 months^(2, 3)^. Some patients may require alternative means of eternal feeding due to prolonged dysphagia^(4)^. Dysphagia increases the chance of aspiration pneumonia, the mortality rate and morbidity rate but also affects the quality of life. According to a study^(5)^, 45% of patients with dysphagia find eating enjoyable, 41% experience anxiety and panic during mealtimes, whereas one-third of patients avoid eating with others because of dysphagia. VFSS is recommended to rule out swallowing pathophysiology and plan for further intervention and management. It can be performed in medically stable patients who can tolerate the sitting position for a minimum of 30 min and are cognitive intact so that they are able to follow simple commands of the speech and language pathologist (SLPs) who performs the VFSS.

During VFSS, SLP closely observes in the fluoroscopic images the oropharyngeal phase of swallowing and tries to detect any abnormality related to the timing of the bolus, swallowing trigger, airway protection, laryngeal elevation and upper oesophageal sphincter opening. To enable visualisation of these swallowing mechanisms, the patient must eat a variety of palatable radiopaque liquid and solid foods, and barium solutions of various concentrations are typically used as contrast media.

A typical examination starts with the patient seated comfortably while acquiring lateral (LAT) views of the lower head and neck, so that all anatomical structures involved in swallowing are visible on the fluoroscopic monitor screen. The LAT images solve a lot of puzzles regarding how the patient is performing in the oral phase, how the bolus of the contrast media is formed and transported to the oral phase and then to the pharyngeal phase. The SLP looks for any delay in swallow trigger, how are the pharyngeal constrictor muscles operate, and most important, how the hyolaryngeal elevation and excursion are imaged while the patient is swallowing. Post swallowing images are reviewed for residue which give more information to the SLP regarding the muscular strength of all the musculature of the swallowing function, and decision is made if the patient is aspirating. When possible, imaging of the oesophagus is included, to exclude gross oesophageal dysfunction. Furthermore, when significant abnormalities occur, a variety of modifications (therapeutic and compensatory techniques) are applied to determine whether they result in improved safety and efficiency of swallowing.

Anterior-posterior (AP) images are also used to facilitate diagnostic and therapeutic decisions, as for example to compare and distinguish between the weaker side of the right and left pharyngeal muscles and to further explore the bolus flow through the cervical oesophagus. More information about the specifics of the VFSS can be found elsewhere^(6–10)^. It should be also noted that recording of the VFSS exam offers the opportunity for careful review by the SLP but can be also used for patient education or for the communication of findings to other healthcare practitioners^(11)^.

In the present study, the VFSS performed at three facilities of Hamad Medical Corporation (HMC) were analytically studied regarding patient dose related metrics, and the results are compared with those reported in relevant studies found in the international literature. The initial motivation for this study was a request from the senior supervisor of the clinical imaging department to investigate the VFSS regarding the radiological protection of patients and personnel, as at that time, VFSS was a relatively unknown examination, to which they had no well-established prior experience.

## Materials and methods

The HMC has a department of Speech and Language Pathology, and all VFSS studies are performed by certified SLPs. According to data available from the dose management system of HMC [Radiation Dose Monitor (RDM), Medsquare SAS, Paris, France], the main volume of VFSS is performed at two facilities, in both of which there are installed modern digital fluoroscopic systems equipped with flat panel image receptors, henceforth referred to as facility A (Siemens Luminos dRF with over-table tube) and facility B (Siemens Luminos Agile with under-table tube). However, there is another facility (henceforth referred to as facility C), where a certified SLP with more than 10 y of experience, performs VFSS using a Philips MultiDiagnost Eleva fluoroscopy system of older technology (image-intensifier-based fluoroscopy system).

For the digital systems connected to the RDM, all data related to each examination are automatically transmitted to the RDM through the picture archiving and communication system (PACS). The transmitted information includes the patient demographics, procedure and examination protocol identifiers and patient dose-related information like the cumulative (total) fluoroscopy time (FT) and kerma-area product (KAP) values at the end of the examination. Depending on the configuration settings selected for each system connected to the RDM, it is also possible to transmit detailed information about each radiation event, and therefore the exposure factors (i.e. the tube potential (kVp), tube current, exposure time, pulse rate for pulsed fluoroscopy, filtration used, etc.) for each fluoroscopic sequence or radiographic acquisitions are individually reported. These data can be reviewed directly in the RDM workstation or exported to an Excel file for further processing and analysis.

In order to investigate the patient radiation dose during VFSS, all data stored in the RDM system regarding all fluoroscopic examinations performed in all HMC facilities for a period of 15 months (1 January 2021–31 March 2022) were exported to an Excel file. All the names of patients, requesting physician, operator and performing physician were de-identified, following the internal regulations for handling patient-related data. VFSS were then filtered from the rest fluoroscopic procedures using the ‘Procedure’ and ‘Study Description’ fields as identifiers, and only those examinations where both these fields were equal to ‘Speech video fluoroscopy’ were selected.

In facility A, where each examination was comprised by more than one data rows in the RDM, a maximum of 35 rows for a single VFSS examination was observed. Successive rows correspond to successive radiation events, but for any given examination, all the rows have in common the cumulative KAP and FT values. The data from each individual sequence are useful to investigate how parameters like the kVp and pulse rate vary during each examination. However, for determining the statistical descriptors of the cumulative FT and KAP values for the specific examination sample (i.e. facility A), it was necessary to filter these data, to avoid the inclusion of the same examination more than once. This was achieved using the data field named ‘Acq. Number’ and selecting only the rows with a value equal to 1. For facility B, data for each examination was limited to a single row, so such a problem did not exist. It must be noted that although the examination images are not stored in the RDM system, it is possible to review anyone of the image series stored in the PACS from the RDM workstation, using the available links.

For facility C, which is not connected to the RDM, the collection of data was made retrospectively from the SLP performing these examinations, for a period of 9 months (1 July 2021–31 March 2022). The examination date, the patient sex and age, the examination protocol used (including the pulsed fluoroscopy rate and additional filter used and typical kVp and mA values), the number of acquisitions and the cumulative values of KAP and FT were extracted from the PACS and manually recorded on a form.

Although this study was focused on the patient doses, a team of medical physicists observed five VFSS procedures performed in facility C, to understand how this procedure is performed and asses the radiation risk involved for the SLP and the radiation technologist performing this examination, monitor the use of protective equipment and interview the SLP in terms of his satisfaction regarding the image quality levels. Using a water phantom to simulate the examination in terms of exposure factors and a dosemeter (RaySafe X2 with scatter probe) for measuring the secondary radiation dose rate at the position where the SLP typically stands during fluoroscopy, the average SLP dose per KAP unit at the eye level was estimated.

The present study was reviewed and approved by the Institutional Research Board of HMC which waived the requirement of patient informed consent. Statistical analysis of the data and statistical comparisons between groups in terms of patient dose related metrics were performed using the IBM SPSS software package. ANOVA test was used to compare the means of the five groups studied, and for pair-wise comparisons, the significance level was set at *p* ≤ 0.05.

## Results

The sample collected comprised by 268 VFSS, 123 in facility A, 108 in facility B and 37 in facility C. However, it was noted that except from facility C where all patients were adult, in facilities A and B, 39% and 90% of the patients, respectively, were children, ranging in age from newborn to 16 y old. The non-adult patients were separated from the adult patient sample which was thus reduced to 123 patients (78% male, 22% female), that is, 75, 11 and 37 adult patients in facilities A, B and C, respectively. The non-adult patient data were analysed separately, both in terms of patient dose related metrics but also in terms of examination purpose, to identify if these examinations were correctly classified as VFSS or whether the procedure ‘Speech video fluoroscopy’ was falsely used.

**Figure 1:**
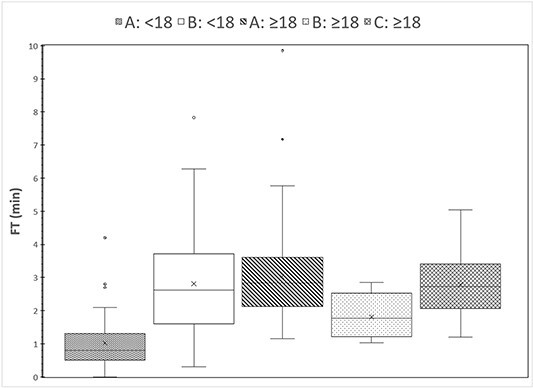
Distributions of cumulative fluoroscopy time values in the five patient groups that underwent VFSS in the three facilities.

**Figure 2:**
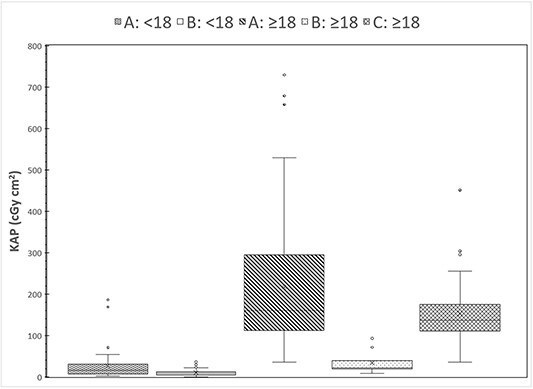
Distributions of cumulative KAP values in the five patient groups that underwent VFSS in the three facilities.

The statistical analysis of cumulative FT and KAP values are tabulated in Table 1, while a graphical description of the distribution of these values of the individual samples is shown in Figures 1 and 2, respectively. It can be seen that for the adult patient samples, FT of facilities A and C are similar (*p* = 0.27) and both higher than B (*p* < 0.05), while KAP values of facility A are larger than those of facility C (*p* < 0.05) and much larger than those of facility B (*p* < 0.001). It should be noted that no correlation of KAP with fluoroscopy time (*R*^2^ < 0.32) or age (*R*^2^ < 0.38) was observed, either for the whole sample or for the samples for each group.

**Table 1:** Statistical analysis of FT and KAP data of the adult and child samples from three facilities.

		***Fluoroscopy time (min)***				**KAP (cGycm** ^**2**^ **)**			
**Facility**	**Patient Nr.**	**Min**	**Median**	**Mean**	**Max**	**Min**	**Median**	**Mean**	**Max**
A (≥18 y)	75	1.2	2.8	3.0	9.9	35.3	160.1	216.5	729.1
B (≥18 y)	11	1.0	1.8	1.8	2.9	8.2	21.7	33.6	92.9
C (≥18 y)	37	1.2	2.7	2.8	5.1	35.3	136.1	152.8	451.0
**All adults**	**123**	**1.0**	**2.7**	**2.8**	**9.9**	**8.2**	**143.5**	***181.0***	**729.1**
*A (<18 y)*	*48*	*0.2*	*2.2*	*2.2*	*6.2*	*2.0*	*16.3*	*26.7*	*186.1*
*B (<18 y)*	*97*	*0.3*	*2.6*	*2.8*	*7.8*	*0.1*	*8.0*	*9.6*	*36.2*
***All children***	***145***	***0.2***	***2.4***	***2.6***	***7.8***	***0.1***	***9.2***	***15.3***	***186.1***

The reason why facility B presents so much smaller KAP values cannot be explained based on exposure factor analysis, as for facility B, a single line per VFSS examination is recorded. Thus, there is no information available for the pulsed fluoroscopy rates used in facility B which could possibly explain the smaller KAP values. Also, for both A and B, no information is transmitted to the RDM regarding the field of view used, which affects the KAP value.

In facility A, from a total of 1717 radiation events, 1202 (70%) were related to adult patients and the rest 515 (30%) to paediatric patients. For the adult patients, in 96% of the radiation events the pulsed fluoroscopy rate of 15 pps was used, but pulsed fluoroscopy rates of 30, 7.5 and 3 pps were also observed. In 85% of the events, a 0.2 mm Cu filter was used while in the remaining 15%, 0.3 mm Cu was used, except from a few radiographic acquisitions (<1%) where no additional filter was used. For paediatric patients, in 73% of the radiation events, the pulsed fluoroscopy rate of 7.5 pps was used and in 16% a pulse rate of 15 pps, but pulse rates of 10 and 3 pps were also observed. In 62% of the events, a 0.2-mm Cu filter was used, whereas in the remaining 38%, 0.3-mm Cu was used, except from a few radiographic acquisitions (<1%) where no additional filter was used. In facility C, the normal field of view is routinely used as the SLP does not use the zoom modes. A pulsed fluoroscopy rate of 15 pps was used in 95% of the cases, 8 pps in 5% of the cases, whereas in 2% of the cases, both 15 and 30 pps rates were used, and additional filtration of 0.2 mm Cu + 1 mm Al. It should be noted that the KAP metres of all these fluoroscopy systems have been checked in the context of routine quality control test, and their accuracy has been found within the accepted limits (within ±25% for the full range of available kVp values).

A possible explanation of whether a statistical difference in KAP is due to the larger fluoroscopy time, the exposure factors (mainly kVp, mAs per pulse and pulsed fluoroscopy rate), the field size or a combination of those can be derived by the comparative study of cumulative FT, KAP and cumulative incidence air kerma (IAK) values to a reference point. The cumulative IAK value at the reference point is measured by KAP metres that have incorporated an ionisation chamber in the centre of the field or most often is calculated from the exposure factors. The reference point is not standard in all radiology units, but usually for under-couch fluoroscopy units, it is at a distance 1 cm above the patient table or for over-couch at 30 cm for the image receptor surface^(11)^. This value is supposed to represent the incident air kerma to the skin of the patient and not the entrance skin dose, as it does not include the backscatter factor, which depends on the beam quality and the radiation field size at the patient entrance surface^(11)^. However, as in VFSS and most fluoroscopic examinations, the patient entrance surface changes, cumulative IAK is not the same as the peak skin dose and for this reason is not routinely reported along with the KAP value.

IAK and KAP should be linearly correlated when the radiation field remains constant. A strong correlation between KAP and IAK was observed for facility A (*R*^2^ = 0.88), while for the other two the correlation was weaker (B: *R*^2^ = 0.64, C: *R*^2^ = 0.68). If now two groups have similar FT and IAK values (i.e. they are no statistical significant differences), but the mean KAP value in one group is statistically significantly different from the other group, it can be assumed that this difference is due to the larger radiation fields used in the group with the larger KAP values and not to a difference in exposure factors, as this should also affect IAK. Similarly, if two groups have largely different IAK values but similar KAP values, then it can be deduced that in the group with the largest IAK values have been used larger exposure factors but smaller field sizes. If now two groups have statistically different fluoroscopy times and both IAK and KAP values are similar, it may be assumed that both the exposure factors and the field size will be larger in the groups with the smaller fluoroscopy time. Of course, in some cases, the relative differences between groups can be such that the relationship between exposure factors and radiation field sizes cannot be postulated.

**Figure 3:**
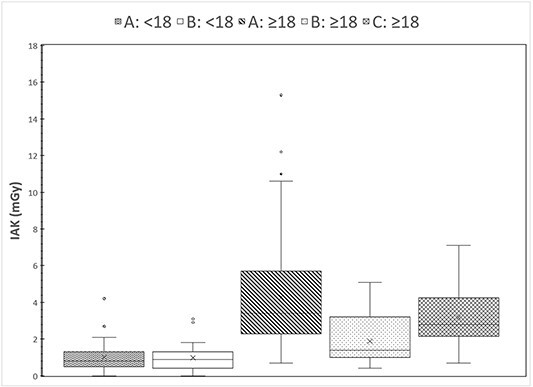
Distributions of cumulative IAK values in the five patient groups that underwent VFSS in the three facilities.

The distribution of cumulative IAK values is shown in Figure 3, and the results of statistical comparisons between FT, IAK and KAP are given in Tables 2–4, respectively. Tables 5 and 6 show the possible differences based on the comparisons regarding the exposure factors (Table 5) and the radiation field size (Table 6). To understand how these comparisons were made, an example is given: group B:<18, compared with group A:<18, has an FT significantly larger, but IAK values are similar, suggesting that in group B:<18 lower exposure factors were used. As group B:<18 has KAP value significantly smaller than group A:<18, despite the similar IAK values, it is deduced that the radiation field values are also smaller. Similarly, the same is true for the radiation field of group B:>18 compared with the groups A:>18 and C:>18. The smaller radiation field used in facility B in comparison with facility A was verified by reviewing three examinations of each group for measuring the radiation field size used. The group A:>18 compared with the group A<:18 according to Table 5 has similar exposure factors. Analysis of the available data on group A exhibited that the mean exposure factors per fluoroscopic frame for group A:<18 were 70.5 kV, 19.2 mA and 5.2 ms (0.1 mAs), whereas the mean exposure factors per fluoroscopic frame for group A:>18 were 72.3 kV, 17.8 mA and 6.1 ms (0.11 mAs), which are similar though a little larger for the adult group.

**Table 2:** Statistical comparison of patient groups regarding FT. In *italics* are given comparisons that involve paediatric patients and in bold the statistically significant differences. *P*-values smaller the 0.001 are denoted as < 0.001.

Facility: group	*A: < 18*	*B: < 18*	A: ≥18	B: ≥18	C: ≥18
*A: < 18*	*1.00 *	***0.014***	***0.001***	*0.120*	***0.03***
*B: < 18*	***0.01***	*1.00 *	*0.30*	***<0.001***	*0.90*
A: ≥18	***0.001***	*0.30*	1.00	**<0.001**	0.27
B: ≥18	*0.12*	***<0.001***	**<0.001**	1.00	**<0.001**
C: ≥18	***0.03***	*0.90*	0.27	**<0.001**	1.00

**Table 3:** Statistical comparison of patient groups regarding IAK. In *italics* are given comparisons that involve paediatric patients and in bold the statistically significant differences. *P*-values smaller than 0.001 are denoted as < 0.001.

Facility: group	*A: < 18*	*B: < 18*	A: ≥18	B: ≥18	C: ≥18
*A: < 18*	*1.00 *	*0.147*	***<0.001***	*0.078*	***<0.001***
*B: < 18*	*0.15*	*1.00 *	***<0.001***	*0.16*	***<0.001***
A: ≥18	***<0.001***	***<0.001***	1.00	**<0.001**	**0.01**
B: ≥18	*0.08*	*0.16*	**<0.001**	1.00	**0.02**
C: ≥18	***<0.001***	***<0.001***	**0.01**	**0.02**	1.00

**Table 4:** Statistical comparison of patient groups regarding KAP. In *italics* are given comparisons that involve paediatric patients and in bold the statistically significant differences. *P*-values smaller than 0.001 are denoted as < 0.001.

Facility: group	*A: < 18*	*B: < 18*	A: ≥18	B: ≥18	C: ≥18
*A: < 18*	*1.00 *	***0.002***	***<0.001***	*0.470*	***<0.001***
*B: < 18*	***0.002***	*1.00 *	***<0.001***	***0.01***	***<0.001***
A: ≥18	***<0.001***	***<0.001***	1.00	**<0.001**	**0.005**
B: ≥18	*0.47*	***0.01***	**<0.001**	1.00	**<0.001**
C: ≥18	***<0.001***	***<0.001***	**0.005**	**<0.001**	1.00

**Table 5:** Postulated statistical difference between groups regarding exposure parameters (kVp, mAs per pulse and pulse rate), based on the combined differences observed between FT, KAP and IAK values. The groups in the column headers are compared with the groups in the first column.

Facility: group	A: < 18	B: < 18	A: ≥18	B: ≥18	C: ≥18
A: < 18		lower	similar	similar	similar
B: < 18			higher	higher	higher
A: ≥18				similar	similar
B: ≥18					similar
C: ≥18					

**Table 6:** Postulated statistical difference between groups regarding the radiation field size, based on the combined differences observed between FT, KAP and IAK values. The groups in the column headers are compared with the groups in the first column. The question mark symbol denotes differences not expected to be statistically significant.

Facility: group	A: < 18	B: < 18	A: ≥18	B: ≥18	C: ≥18
A: < 18		smaller	similar	smaller	similar
B: < 18			larger?	larger?	larger?
A: ≥18				smaller	similar
B: ≥18					larger
C: ≥18					

The comparison of the statistical values for FT and KAP regarding the samples of all adults and all paediatric patients included in this study, with relevant data from the international literature is shown in Table 7. It can be seen that for adult patients, the FT and KAP values are within the range of the values reported in the literature, and they are the third lowest of all the values reported in Table 7. Regarding children, the KAP values observed in the present study are more than 10 times smaller than the values found in the literature, though the mean FT is similar to the values reported in the literature.

**Table 7:** Comparison of KAP and fluoroscopy time data statistics of this study with respective values from relevant studies in the international literature.

		***Fluoroscopy time (min)***				**KAP (cGycm** ^**2**^ **)**			
	**Source**	***Min***	***Median***	***Mean***	***Max***	**Min**	**Median**	**Mean**	**max**
**This study (only adults)**	**123**	**1.0**	**2.7**	**2.8**	**9.9**	**8.2**	**144**	**181**	**729**
Wright *et al.*^(12)^	23	0.5	-	4.8	8.3	28	-	400	974
Chan *et al.*^(13)^	17	-	-	18.0	-	258	-	842	2151
Crawley *et al.*^(14)^	21	2.5	3.7	-	4.3	310	350	-	520
Moro *et al.*^(15)^	22	1.4	-	2.6	5.1	100	-	230	540
Zammit-Maempel *et al.*^(16)^	230	0.3	2.9	3.0	9.4	5	140	160	1000
Chau and *Kung*^(17)^	398	-	-	4.2	-	-	-	242	-
Kim *et al.*^(18)^	271	-	-	3.37	-	-	-	994	
Bonilha *et al.*^(19)^	200	0.4	-	2.6	6.45	17	-	134	594
***This study (only children)***	***145***	***0.2***	***2.4***	***2.6***	***7.8***	***0.1***	***9.2***	***15.3***	***186.1***
Kim *et al.*^(18)^	*24*	*-*	*-*	*2.42*	*-*	*-*	*-*	*371*	
Ko *et al.*^(20)^	*89*	*-*	*-*	*2.24*	*-*	*-*	*-*	*241*	*-*

From the five VFSS observed, it was understood that the SLP is routinely wearing protective equipment (lead apron and collar but not lead glasses), as during the procedure, he stays within the examination room and relatively close to the patient (about 1 m away). The SLP did not report any problems regarding the image quality of the image quality, though pulsed fluoroscopy of 15 pps is routinely used instead of the maximum available pulse rate of 30 pps.

According to simulation measurements, using typical exposure factors (15 pulses/s, 73 kV, 18 mA, 7.1 ms per pulse, normal field of view and 0.2 mm Cu additional filtration), the average secondary radiation dose rate at the SLP position is 5.7 μGy/min. Thus, given that the average FT and KAP values per examination for adult patients are 2.8 min and 181 cGycm^2^, the dose to the eyes of the SLP per examination will be about 16 μGy or 0.09 μGy/cGycm^2^. Therefore, a SLP would have to perform about 1250 VFSS to reach the maximum limit of 20 mSv per year for the eye, which is much larger than the current workload (≤100 patients per year). Regarding the radiographer, VFSS do not entail any radiation risk, as during the procedure, he/she remains within the control console room.

## Discussion

Like all examinations involving ionising radiation, VFSS entail a certain radiation risk for the patient but also for the SLP performing the examination, as the SLP must remain within the room and close to the patient, to administer the contrast media necessary for the examination and instruct the patient to perform the swallowing tasks required. Regarding the radiation risk to patients, there are several studies in the international literature investigating the radiation dose levels to patients from VFSS, the results of which are summarised in Table 7. According to Palmer *et al*.^(7)^, for patients who chew and swallow normally, VFSS requires about 2–3 min of actual fluoroscopy, whereas for patients who chew and swallow food very slowly, the study is longer. Some patients may require extensive empirical testing of therapeutic and compensatory manoeuvers under fluoroscopy. For adult patients, the average KAP value is rather low and indicates that VFSS are highly unlikely to entail a significant risk to patients.

Regarding effective dose (*E*) to adult patients, Crawley *et al*.^(14)^, who reported a median KAP value more than twice the median value of the adult patients of the present study, also reported an estimated median *E* value of 0.85 mSv (range: 0.76–1.3 mSv). From these values, it can be derived that the KAP to *E* conversion coefficient in that study was 0.00243 mSv/cGycm^2^. Crawley *et al*.^(14)^ also reported a median equivalent dose to the thyroid of the patient of 13.9 mSv (range 12.3–20.7 mSv), which, assuming a backscatter factor of 1.3, results in an IAK of about 10.7 mGy. This value is slightly more than twice the third quartile value of IAK that was observed for the adult sample of the present study (4.9 mGy). Zammit-Maempel *et al*.^(16)^, which reported FT and KAP values similar to the present study, also reported an estimated median *E* value of 0.2 mSv (range: 0.01–1.4 mSv). Therefore, it can be derived that the KAP to *E* conversion coefficient in that study was 0.00143 mSv/cGycm^2^, almost half the value derived from the Crawley *et al.*^(14)^ data. Chau and Kung^(17)^ reported an estimated median *E* value of 0.31 mSv (derived KAP to *E* conversion coefficient 0.00128 mSv/cGycm^2^), and Kim *et al*.^(18)^, reported an estimated median *E* value of 1.23 mSv (derived KAP to *E* conversion coefficient 0.00128 mSv/cGycm^2^). Using the highest value of KAP to *E* conversion coefficient derived from the data found in the literature for adults, a median *E* value of 0.35 mSv is estimated for the adult group of the present study.

Regarding the VFSS performed in children, the large number of cases identified came as a surprise, as initially was expected that VFSS is most frequently used in adults, and especially of large age. However, for the adult group, the range of ages was 19–86 y with a mean of 54 y, and for the paediatric group the range of ages was from 0 to 16 y with a mean of 1.8 y. What it was most striking was that 65 cases were identified where the child’s age was 1 month or less. For this reason, VFSS performed in paediatric patients were also included in this study.

VFSS is indicated for children when there is a concern related to child’s swallows, airway protection during swallowing, chronic respiratory problems including chest infections/pneumonia, coughing during meals and frequent choking. By reviewing the images of a few of the newborn patient cases, there were identified radiation fields as small as 6 cm × 6 cm (just the area around the cervix and the mouth was imaged), and as large as 15 cm × 15 cm (almost the whole baby’s anatomy, from the forehead to xiphisternum was imaged). This was indicative of the great diversity encountered regarding the radiation field size selection strategies, which could not be further analysed, given the fact the field size data are not transferred to the RDM. Regarding effective dose (*E*) to paediatric patients, Kim *et al.*^(18)^ reported a mean *E* of 0.48 mSv (derived KAP to *E* conversion coefficient 0.00129 mSv/cGycm^2^). Ko *et al.*^(20)^ reported a mean *E* of 0.29 mSv (derived KAP to *E* conversion coefficient 0.0012 mSv/cGycm^2^). Using the highest value of KAP to *E* conversion coefficient derived from the data found in the literature for paediatric patients, a median *E* value of 0.02 mSv was estimated for the children group in the present study.

In the present study, it was seen in the majority of VFSS for adult patients, the pulse rate of 15 pulses per second (pps) was used, while in most of the paediatric patients, a pulse rate of 7.5 pps was preferred. The debate regarding the use of pulse (or frame) rates lower than 30 pps is summarised in the study of Hong *et al*.^(21)^. The authors concluded that while lowering the dose of pulse rate is an obvious dose reduction strategy, it is not justified, given the limited radiation risks associated with VFSS and the negative effects that may have on the diagnostic accuracy, as it may result in a loss of diagnostic information. However, as in our study pulse rates of 15 pps or lower are commonly used, it can be deduced that the SLPs performing the VFSS in our facilities do not share this opinion.

Regarding the radiation risk to the SLPs, Crawley *et al*.^(14)^ reported that the total whole-body dose for 21 procedures, as reflected by the dosemeter worn under the lead apron, was less than 0.3 mSv (the dose for each of the three consecutive dosemeters worn during the period of the study was below the reporting threshold of 0.1 mSv). Mean operator doses were 0.5- (eyes) and 0.9-mSv equivalent dose (hands). In the study by McLean et al.^(22)^, the average dose per procedure measured to the thyroid of the SLPs in three facilities was, respectively, 17, 3.2 and <6 μGy over the thyroid shield (lead collar), whereas below the shield the dose was below the detection limit of 6 μGy in all three facilities. The dose of 17 μGy at the thyroid per procedure is very close to the value of 16 μSv per procedure at the eye level estimated in the present study. The dose at the eye level is more important than the dose to thyroid, as the eyes are usually not protected unlike thyroid, and the limit to the eyes has been reduced to 20 mSv per year.

## Conclusion

For adult patients, the FT and KAP values are found within the lower range of the values reported in the literature. Therefore, this indicates that VFSS are performed by well-trained health professionals who use the fluoroscopy mode with caution to protect both patients and themselves. At the observed dose levels, VFSS is highly unlikely to entail any significant risk to patients or to the SLPs regarding the occurrence of deterministic effects, and the only concern is the small risk for the occurrence of stochastic effects (radiogenic cancer).

## Data availability

Data may be available on request.

## Funding

Open Access funding provided by the Qatar National Library.
